# Supplementary material for: The dynamics of γδ T cell responses in nonhuman primates during SARS-CoV-2 infection
Source: Commun Biol. 2022 Dec 16;5:1380. doi: 10.1038/s42003-022-04310-y (PMC9756695; doi:10.1038/s42003-022-04310-y)
Supplement: Supplementary file 2 — Supplementary Information [file 42003_2022_4310_MOESM2_ESM.pdf]

Supplementary Information

Manuscript Title: The dynamics of γδ T cell responses in nonhuman primates during SARS-CoV-2 infection

Authors: Alyssa C. Fears[1], Edith M. Walker[1], Nicole Chirichella[1], Nadia Slisarenko[1], Kristen M. Merino[1], Nadia Golden[1], Breanna Picou[4], Skye Spencer[4], Kasi E. Russell-Lodrigue[3], Lara A. Doyle-Meyers[3], Robert V. Blair[2], Brandon J. Beddingfield[1], Nicholas J. Maness[1,5], Chad J. Roy[1,5], Namita Rout[1,5,6]*

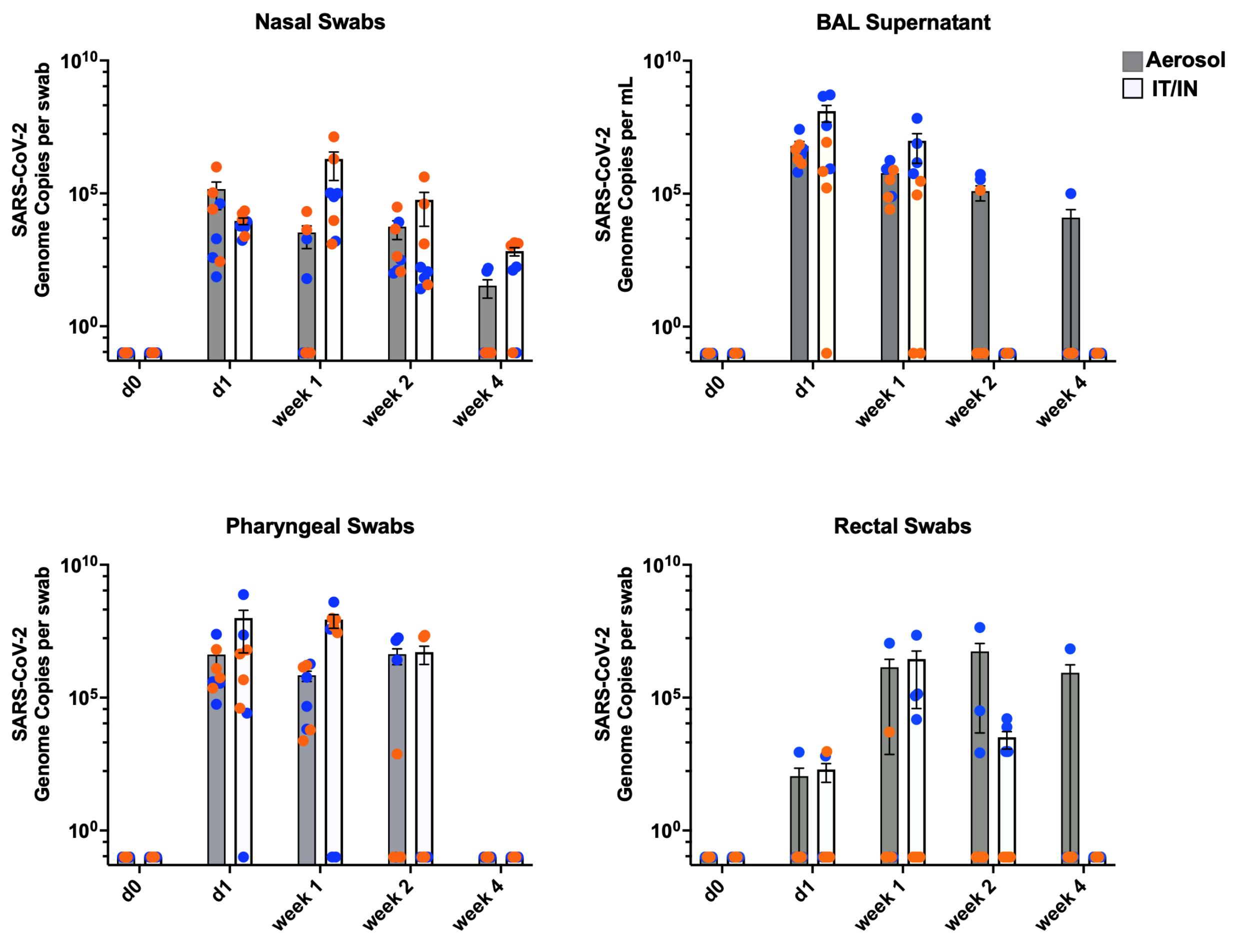

Supplementary Fig. 1. Viral loads at day 1, week 1, week 2, and week 4 assessed in BAL supernatant and nasal, pharyngeal, and rectal swabs via RT-qPCR for genomic RNA (n=8/group). Individual values shown for each animal as filled circles in orange for RMs (n=4/route) and blue for AGMs (n=4/route).

	Aerosol								IT/IN							
	Rhesus				African Green				Rhesus				African Green			
DPI	IR12	IJ01	KN90	JG28	NB86	NB78	NB76	NC06	JK41	KH77	KT88	LP05	NB77	NC04	NC07	NB81
2														+		
3									+		+		+	++		
4														++	+	
5											++					
6																
7									+		+	+	+ ^	++	+	+
8													+	++ *		+
9										+		+		+++ *		+
10												+		+++	+	
11						++					++		++	++		
12						++			+				++	++		
13						++	++		+					++ **		++
14		+				+			+		+	+	+	+	+++ ^	
15		+							++		++	++		++ *	++	
16		+				+	+		+		+	++	+	+		
17		+				++					++		+	+	++	
18		+									++			++		
19		++				++	++		++		++	++		++		
20		+				+	+				++			++		
21		+	++	++		++					++	++		++ *	++	
22		++							++		++		++	++	++	
23		+				+			++		++	++	++	++ *	++	
24		++				+	+				++	++	++	++	++	
25		++		++		++					++	++	++	++	++	
26		++				++			++		++	++	++	++	++	
27		++	++			++	++		++			++		++		
28		++	++				++		++			++	++			
29									++			++				
30		++							++			++				

* refusal to move around cage

^ supplemental oxygen received

Supplementary Fig. 2. Clinical symptoms in the study animals. Changes in animal behavior and clinical symptoms were recorded by veterinary staff and Veterinarians from day 0 to 1 month (28-30 dpi). The aerosol and IT/IN groups had 8 animals each, with equal distribution of RM and AGM (n=4/species).

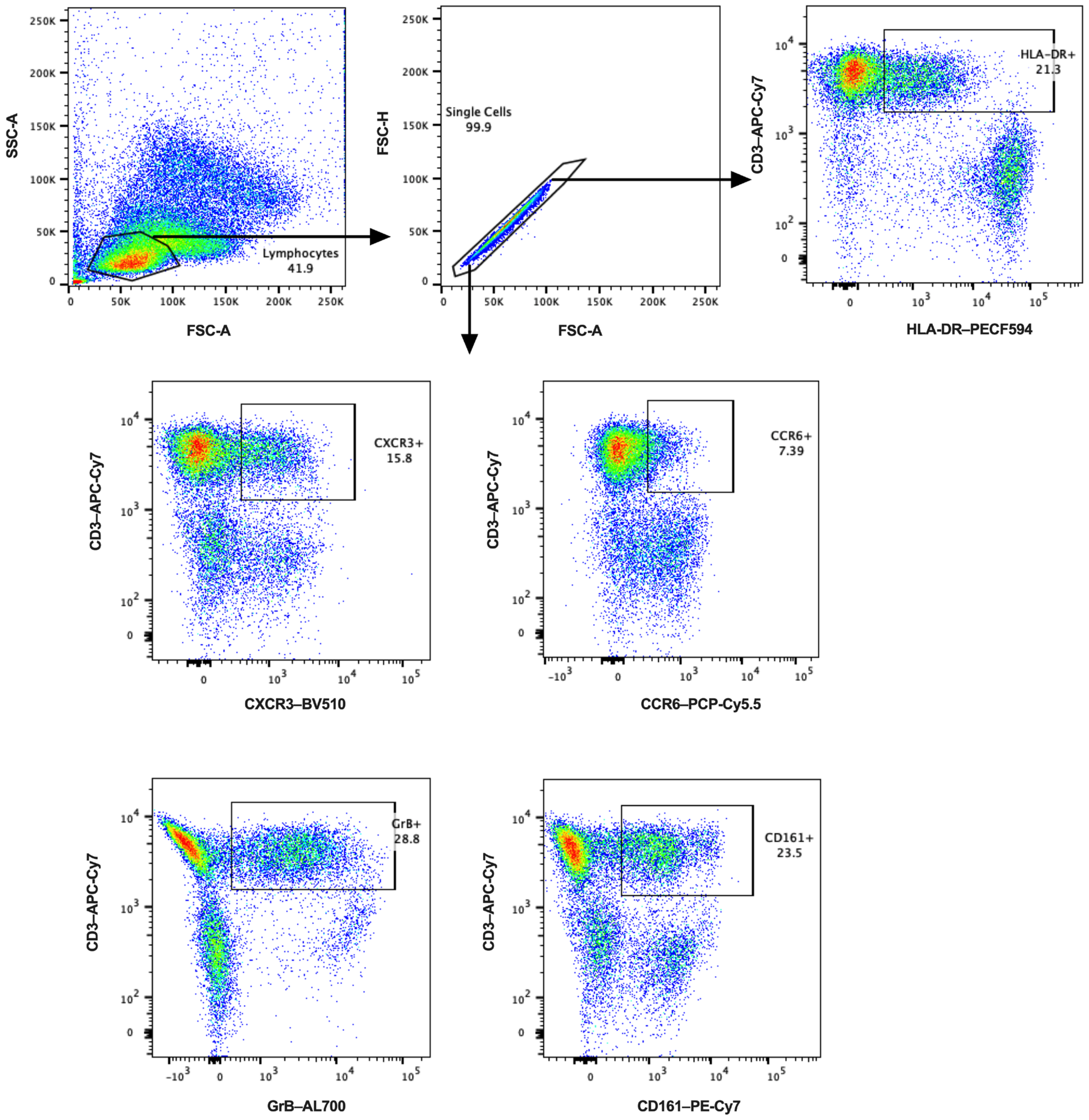

Supplementary Fig. 3. Gating strategy for phenotypic characterization of T cell subpopulations. Representative FACS plots of PBMC showing gating strategy for the expression of HLA-DR, CXCR3, CCR6, GrB, and CD161 on CD3-positive T cells.

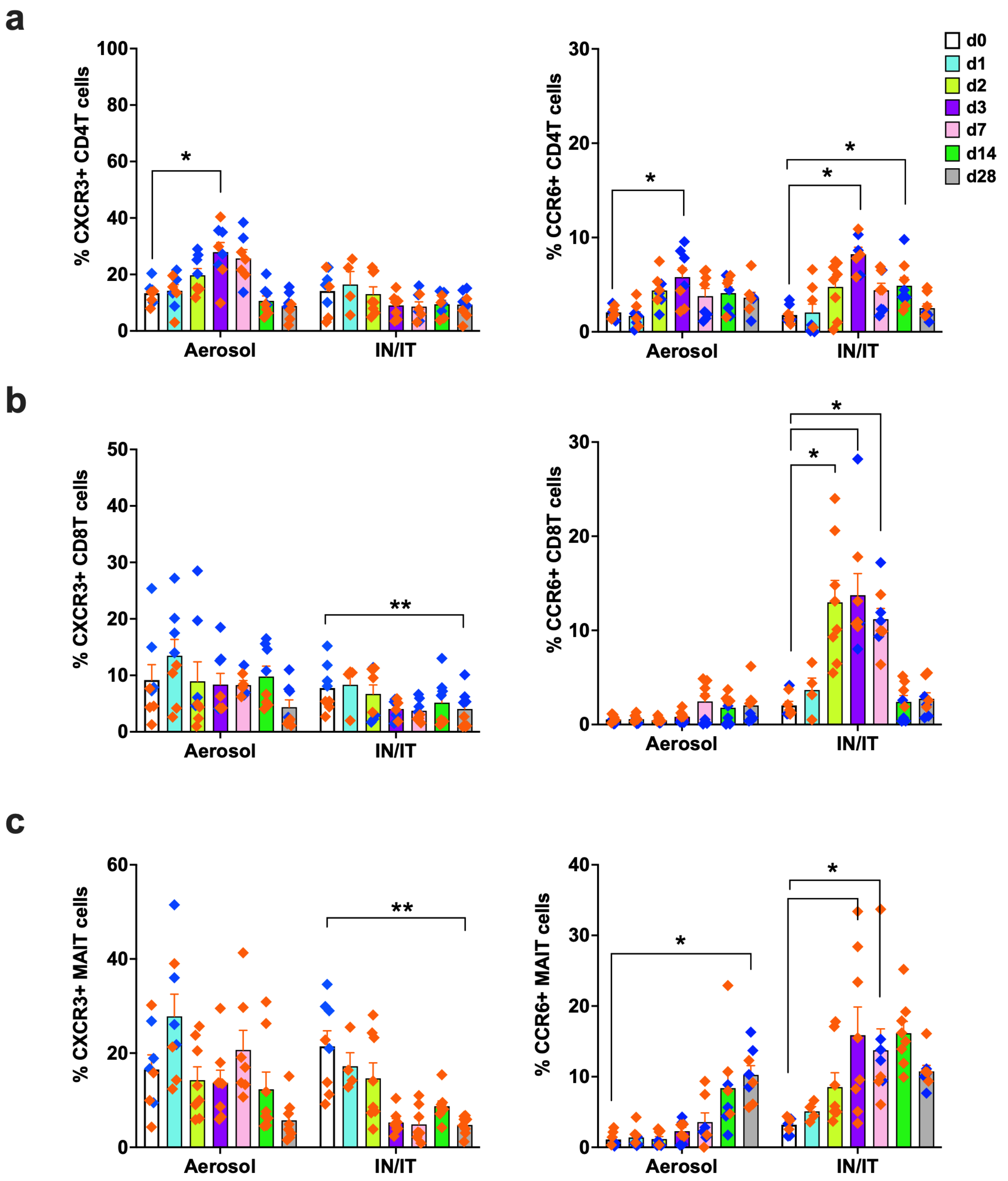

Supplementary Fig. 4. CXCR3 and CCR6 expression by circulating CD4 T, CD8 T, and MAIT cells following aerosol and IT/IN SARS-CoV-2 infection of NHPs. Frequencies of CXCR3+ and CCR6+ cells in (**a**) CD4 T cells, (**b**) CD8 T cells, and (**c**) MAIT cells in PBMC of

aerosol versus IT/IN exposure groups of RMs (orange diamonds) and AGMs (blue diamonds) through the course of one month SARS-CoV-2 infection (n=8/group). Graphs show mean and SEM. Comparisons of different time points with respect to day 0 baseline data were done using two-way ANOVA with mixed effects model and Dunnett's post hoc tests. Asterisks indicate significant differences between time points (*$p < 0.05$; **$p < 0.01$).

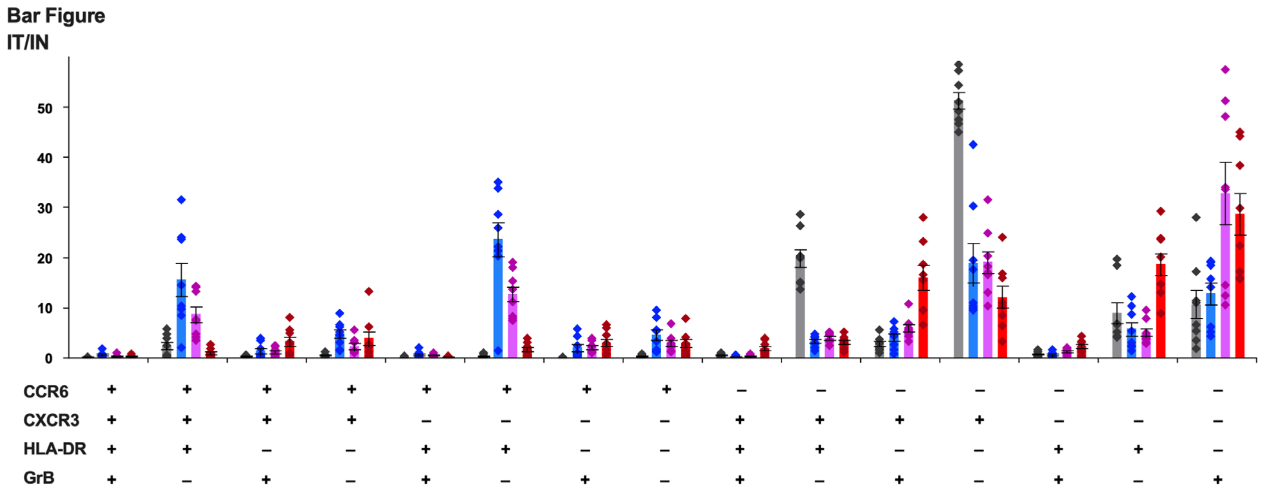

Wilcoxon Rank Sum Test
IT/IN
Category

CCR6	CXCR3	HLA-DR	GrB	3 dpi	7 dpi	14 dpi
+	+	+	+	0.0054	0.0742	0.0460
+	+	+	-	0.0033	0.0063	0.5286
+	+	-	+	0.0117	0.0063	0.0117
+	+	-	-	0.0008	0.0011	0.0033
+	-	+	+	0.0087	0.0023	0.4309
+	-	+	-	0.0008	0.0008	0.0136
+	-	-	+	0.0008	0.0008	0.0008
+	-	-	-	0.0008	0.0008	0.0019
-	+	+	+	0.3720	0.5635	0.0033
-	+	+	-	0.0008	0.0008	0.0008
-	+	-	+	0.0587	0.0074	0.0011
-	+	-	-	0.0087	0.0008	0.0008
-	-	+	+	0.5286	0.0742	0.0033
-	-	+	-	0.5286	0.0520	0.0063
-	-	-	+	0.1152	0.0209	0.0046

Supplementary Fig. 5. Early changes in functional phenotype of blood γδ T cells following IT/IN infection with SARS-CoV-2. Bar graph showing proportion of γδ T cells in PBMC expressing different combinations of CCR6, CXCR3, HLA-DR, and granzymeB at day 0 (grey bar), d3 (blue bar), d7 (pink bar), and d14 (red bar) following IT/IN SARS-CoV-2 exposure (n=8/group)**.** Graph shows mean and SEM. Table underneath shows 3 dpi, 7 dpi, and 14 dpi time points compared to baseline using Wilcoxon matched-pairs signed rank test and statistically significant differences are highlighted in red boxes.

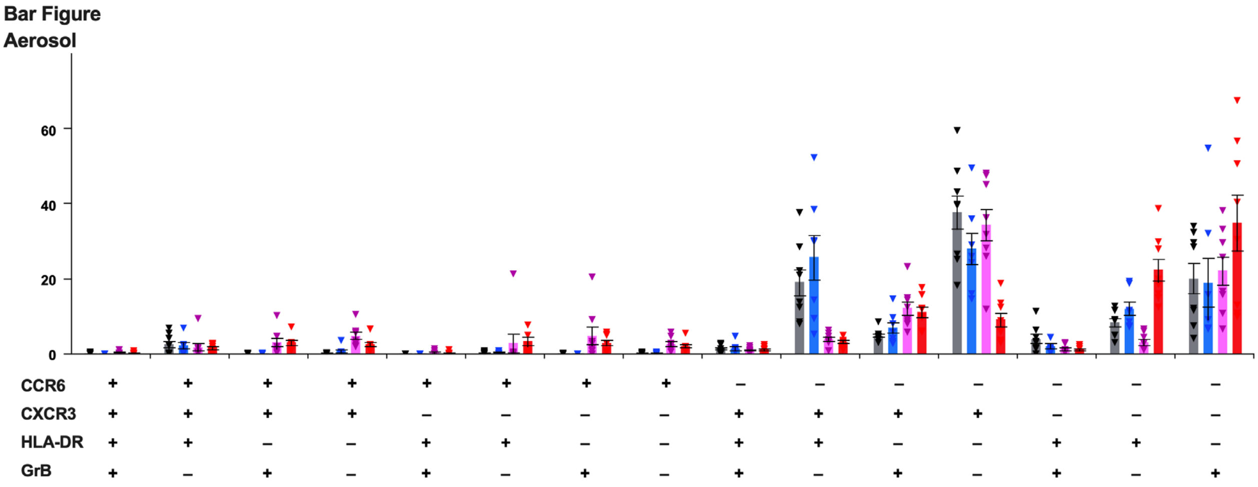

Wilcoxon Rank Sum Test
Aerosol

CCR6	CXCR3	HLA-DR	GrB	3 dpi	7 dpi	14 dpi
+	+	+	+	0.8170	0.0742	0.3720
+	+	+	-	0.8170	0.9581	0.9164
+	+	-	+	0.2030	0.0008	0.0008
+	+	-	-	0.2716	0.0008	0.0008
+	-	+	+	0.3545	0.1278	0.0929
+	-	+	-	0.9079	0.4948	0.0074
+	-	-	+	0.6854	0.0016	0.0008
+	-	-	-	0.6434	0.0008	0.0008
-	+	+	+	0.6434	0.8748	0.4623
-	+	+	-	0.3545	0.0008	0.0008
-	+	-	+	0.2472	0.0011	0.0016
-	+	-	-	0.4875	0.5286	0.0008
-	-	+	+	0.4179	0.0929	0.1036
-	-	+	-	0.0562	0.0209	0.0008
-	-	-	+	0.8170	0.3446	0.0742

Supplementary Fig. 6. Early changes in functional phenotype of blood γδ T cells following aerosol infection with SARS-CoV-2. Bar graph showing proportion of γδ T cells in PBMC expressing different combinations of CCR6, CXCR3, HLA-DR, and granzymeB at day 0 (grey bar), d3 (blue bar), d7 (pink bar), and d14 (red bar) following aerosol SARS-CoV-2 exposure (n=8/group)**.** Graph shows mean and SEM. Table underneath shows 3 dpi, 7 dpi, and 14 dpi time points compared to baseline using Wilcoxon matched-pairs signed rank test and statistically significant differences are highlighted in red boxes.

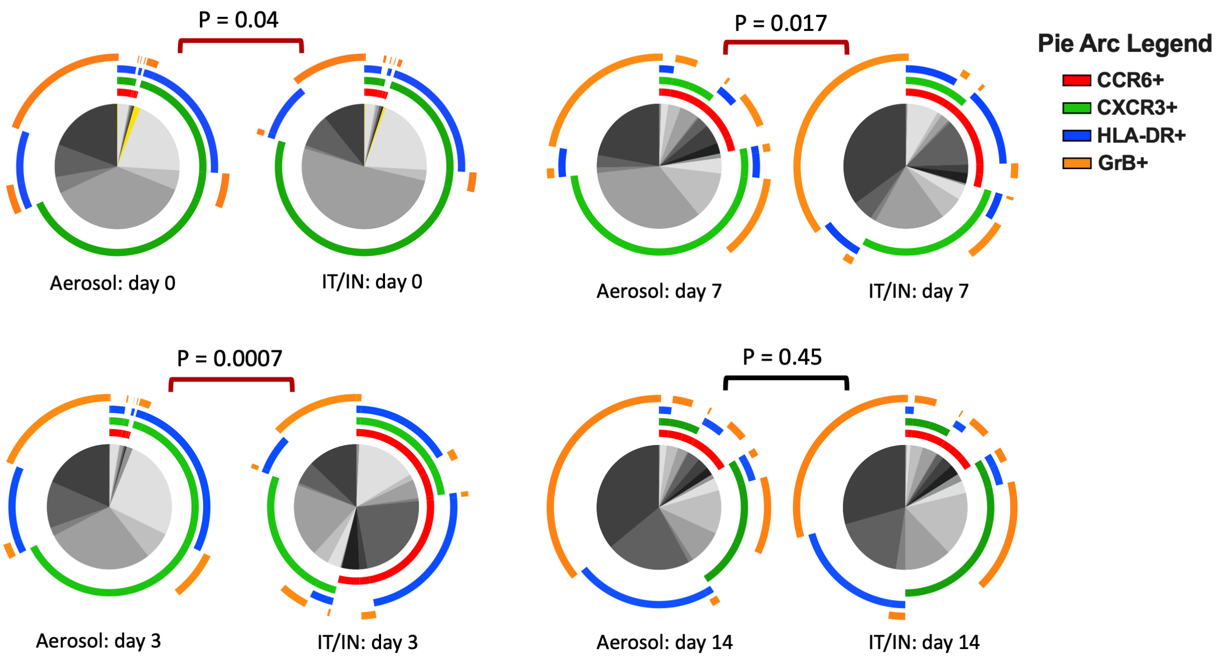

Supplementary Fig. 7. Early induction of distinct polyfunctional phenotypes in circulating γδ T cells between aerosol and IT/IN routes of SARS-CoV-2 infection. Pie charts comparing early changes in polyfunctional γδ T cell phenotype based on the expression of HLA-DR, CXCR3, CCR6, and granzyme B on day 0, day 3, day 7 and day 14 of SARS-CoV-2 infection via aerosol and IT/IN delivery (n=8/group). The arcs around the circumference indicate each marker expressed by the proportion of cells that lie under the arc. HLA-DR is shown in blue, CCR6 in red, CXCR3 in green, and granzymeB in orange. p values were computed using the SPICE permutation test.

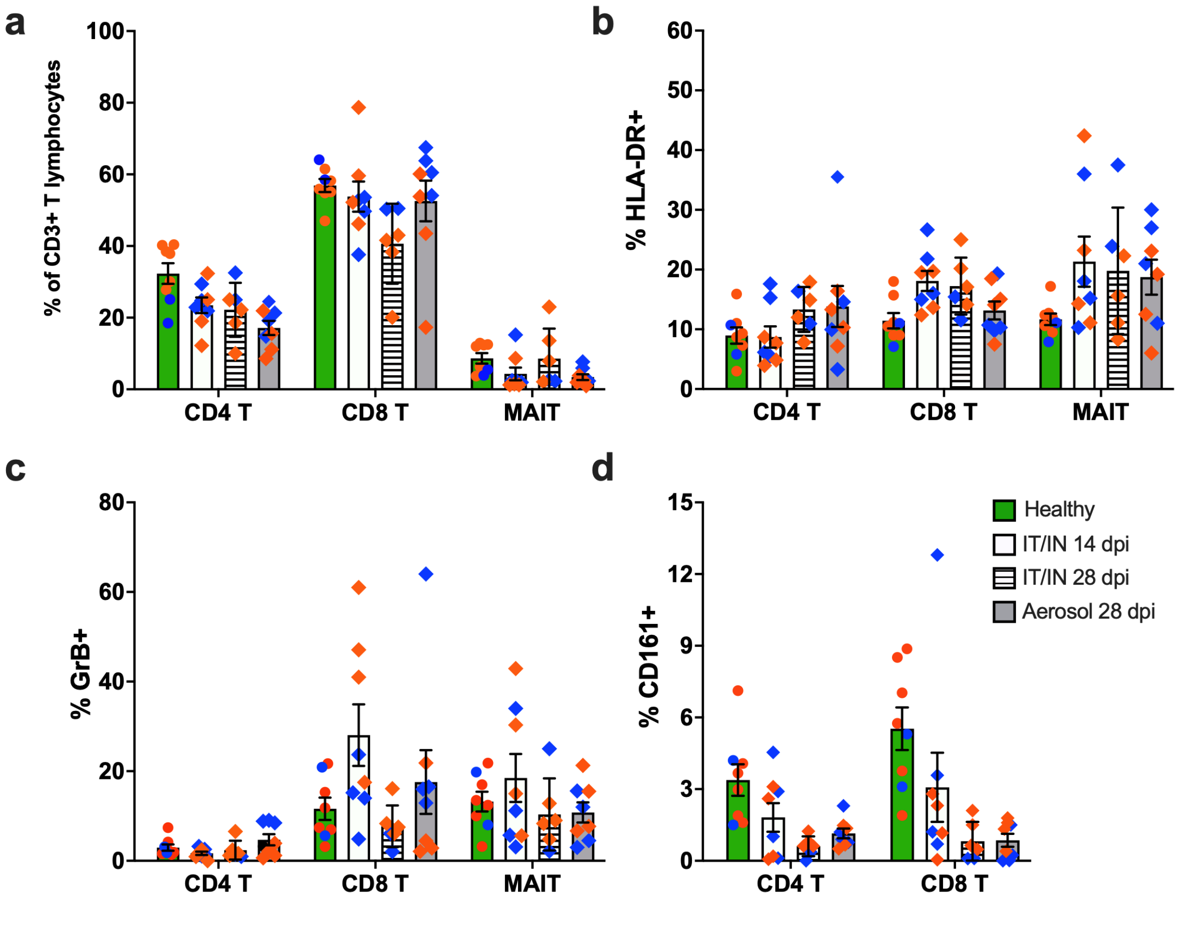

Supplementary Fig. 8. Frequency activation and functional phenotype of BAL CD4, CD8, and MAIT cells during SARS-CoV-2 infection. a Cross-sectional comparison of frequencies of CD4 T cells, CD8 T cells, and MAIT cells in CD3+ T lymphocytes in BAL fluid from healthy RM (orange circles) and AGM (blue circles) with SARS-CoV-2 infected RMs (orange diamonds) and AGMs (blue diamonds) at 14 dpi and 28 dpi via IT/IN, or 28 dpi via aerosol challenge (n=8/group). Expression levels of HLA-DR **(b)**, granzyme B **(c)**, and CD161 **(d)** on total γδ T cells**,** Vδ1 T cells, and Vδ2 T cells in BAL. Graphs show mean and SEM. Healthy and infected animals compared using Mann Whitney test and longitudinal comparisons between 14

dpi and 28 dpi using Wilcoxon matched-pairs signed rank test revealed no significant differences.

Supplementary Table 1. RT-qPCR Primers and Probes.

Target	Primer/Probe Designation	Sequence
Genomic Nucleocapsid	2019-nCoV_N1-F	5’-GAC CCC AAA ATC AGC GAA AT-3’
	2019-nCoV_N1-R	5’-TCT GGT TAC TGC CAG TTG AAT CTG-3’
	2019-nCoV_N1-P	5’-FAM ACC CCG CAT TAC GTT TGG TGG AACC-BHQ-3’
Subgenomic Nucleocapsid	SgN-F	5’-CGA TCT CTT GTA GAT CTG TTC TC-3’
	SgN-R	5’-GGT GAA CCA AGA CGC AGT AT-3’
	SgN-P	5’-56-FAM/TAA CCA GAA/ZEN/TGG AGA ACG CAG TGG G/3IABkFQ/-3’

Supplementary Table 2. Antibodies for Flow Cytometric Analysis

**PBMC Antibodies**				
**Target**	**Flourochrome**	**Vendor**	**Clone**	**Catalog #**
CD14	BV711	BD	MFP9	563372
CD161	PE	BD	HP-3G10	566843
CD196	PCP-Cy5.5	BD	11A9	560467
CD3	APC-Cy7	BD	SP34-2	557757
CD4	BV650	BD	L200	563737
CD8	BV605	BD	SK1	564116
Granzyme B	AF700	BD	GB11	560213
HLA-DR	PE-CF594	BD	G46-6	562304
CD183	BV510	Biolegend	G025H7	353726
CD20	BV711	Biolegend	2H7	302342
CD25	PE-Cy5	Biolegend	BC96	302608
TCR γδ	PE-Cy7	Biolegend	B1	331222
TCR Vα7.2	BV421	Biolegend	3C10	351716
TCR Vδ1	FITC	ThermoScientific	TS8.2	TCR2730
TCR Vδ2	APC	ThermoScientific	15D	TCR1732
**BAL Antibodies**				
**Target**	**Fluorochrome**	**Vendor**	**Clone**	**Catalog #**
CD14	BV711	BD	MFP9	563372
CD3	APC-Cy7	BD	SP34-2	557757
CD4	BV650	BD	L200	563737
CD45	BV510	BD	D058-1283	563530
CD8	BV605	BD	SK1	564116
Granzyme B	PE	BD	GB11	561142
HLA-DR	PE-CF594	BD	G46-6	562304
TCR g/d	PCP-Cy5.5	BD	B1	564157
CD161	PE-Cy7	Biolegend	HP-3G10	339918
CD20	BV711	Biolegend	2H7	302342
CD25	PE-Cy5	Biolegend	BC96	302608
TCR Vα7.2	BV421	Biolegend	3C10	351716
TCR Vδ1	FITC	ThermoScientific	TS8.2	TCR2730
TCR Vδ1	APC	ThermoScientific	15D	TCR1732

Antibody concentrations were used as per manufacturer recommendations.
