# Supplementary material for: The dynamics of γδ T cell responses in nonhuman primates during SARS-CoV-2 infection
Source: Commun Biol. 2022 Dec 16;5:1380. doi: 10.1038/s42003-022-04310-y (PMC9756695; doi:10.1038/s42003-022-04310-y)
Supplement: Supplementary file 3 — Description of Additional Supplementary Data [file 42003_2022_4310_MOESM3_ESM.docx]

**Description of Additional Supplementary Files**

**File name:** Supplementary Data 1

**Description:** The numerical source data in each sheet labelled by relevant Figure numbers.
